# Porcelain Atrium: A Case Report with Literature Review

**DOI:** 10.1155/2011/501396

**Published:** 2011-09-18

**Authors:** Krystle Leacock, Andre J. Duerinckx, Bonnie Davis

**Affiliations:** ^1^Howard University College of Medicine, Washington, DC 20059, USA; ^2^Department of Radiology, Howard University College of Medicine, Washington, DC 20060, USA

## Abstract

Massive left atrial wall calcification, or porcelain atrium, is very rare. We describe a case of an unusual pattern of cardiac calcification demonstrated on routine preoperative chest X-ray for cataract surgery in a 71-year-old Nigerian woman. Past medical history was significant for mitral stenosis and atrial fibrillation. Radiographic imaging revealed curvilinear high density areas of calcification outlining the left atrium on the chest X-ray. Noncontrast CT scan of the thorax confirmed the left atrial distribution of calcification and, thus, the diagnosis of porcelain left atrium.

## 1. Introduction

Massive calcification of the left atrial walls, also known as a porcelain atrium, is a rare condition. Though without immediate clinical sequela, its existence yields important implications in the setting of mitral valve surgery [1]. It is reported infrequently and generally has been found as an incidental radiological finding. On conventional radiographic imaging, the left atrial calcification is described as a high density ring encircling the left atrium. Noncontrast CT imaging of the thorax reveals, in greater detail, deposition of calcification outlining the wall of the left atrium. We present a case of massive left atrial calcification sparing the interatrial septum, found incidentally on routine preoperative chest X-ray in a woman of African descent.

## 2. Case Report

**Figure 1:**
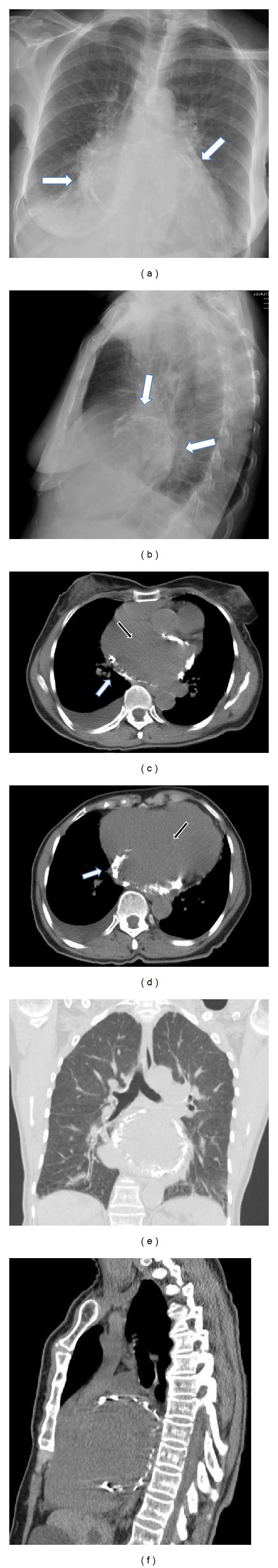
A 71-year-old woman with known mitral stenosis and atrial fibrillation underwent routine preoperative screening for cataract surgery. The chest X-ray showed curvilinear dense calcifications on both the frontal ((a), arrows) and lateral ((b), arrows) views. A follow up noncontrast CT of the chest better localized these calcifications on axial images ((c), (d), white arrows), coronal image (e) and sagittal image (f), as being in the wall of the left atrium. The interatrial septum ((c), black thin arrow) and mitral valve annulus ((d), thin black arrow) were not calcified. The coronal (e) and sagittal (f) images show a pattern of calcification very similar to the one seen on the frontal (a) and lateral (b) chest X-rays. Incidental right pleural effusion and liver cysts were also shown.

A 71-year-old Nigerian female, with past medical history of rheumatic valvular disease with mitral stenosis, and atrial fibrillation, presented for routine preoperative chest X-ray for cataract surgery. The patient was asymptomatic, and otherwise in reasonably good health. A recent echocardiogram confirmed severe left atrial and left ventricular enlargement; the mitral valve area was found to be 0.7 cm^2^ (normal 3-4 cm^2^), consistent with mitral valve stenosis. Incidental findings on the chest X-ray revealed an unusual pattern of curvilinear cardiac calcification (Figures 1(a) and 1(b)). Subsequently, a noncontrast CT scan of the thorax was obtained for further characterization and anatomical localization (Figures 1(c), 1(d), 1(e), and 1(f)). Surgical intervention was offered in years past, though did not occur for unknown reason(s).

## 3. Discussion

 Cardiac and pericardial calcifications have been well described in the literature. Left atrial calcifications are a less commonly encountered subset of these. Calcifications of the left atrium can involve the left atrial appendage, left atrial free wall, or mitral valve apparatus, and in more severe cases it may involve all three sites [1, 2]. Calcification of the left atrium was originally described in 1898 [2] and is commonly known to be associated with long standing rheumatic valve disease. If the calcifications are heavy, then the term “porcelain left atrium” is used. The interatrial septum is often spared. When the interatrial septum is not spared the term coconut atrium [2] is used. A classification scheme for left atrial calcification, denoted as A, B, and C, has been proposed [3]. Type A arises when calcification is confined to the left atrial appendage; the underlying lesion in type A is often mitral stenosis [2]. This type of calcification almost always is associated with thrombus in the appendage. Type B occurs when the free wall of the left atrium and mitral valve are calcified, although the valve calcification is not always appreciated from chest radiographs; this pattern demonstrates advanced mitral stenosis [3]. Type C occurs when a small area of calcification is confined to the left atrium's posterior wall; this is termed McCallum's patch, and it results from a jet lesion because of mitral regurgitation [3]. In our case report, none of the above classifications of the types of left atrial calcifications accurately describe our patient's pattern of left atrial calcification, as the calcification process in our patient did not involve the mitral valve nor the left atrial appendage.

Calcification of the left atrium has a predilection for females with an age range of late 50's to early 60's. Review of the literature revealed that almost all of the patients with left atrial calcification were found to be in chronic atrial fibrillation, and the interatrial septum was uninvolved, as seen with our patient. In addition, these patients are usually found to have undergone at least one previous operation [4], most often a mitral valve operation. 

 Various imaging techniques may be used to aid in the diagnosis of a porcelain atrium. Chest X-ray imaging demonstrates mural calcifications that appear as a thin curvilinear density tracing the outline of the left atrium in part or completely [5]. In addition, Hawthorne et al. [5] best described the appearance of left atrial calcification on PA films as a completely calcified wall which appears as a C-shaped curvilinear density with the opening of the C lying anteriorly in the region of the mitral annulus. Due to the posterior location of the left atrium, transesophageal echocardiography permits imaging of the chamber. However, there is debate as to whether calcification limits the use of this technique. Vilacosta et al. [6] found it to be an inadequate study for patients with left atrial calcification, while Goel and Singh [7] found the technique to be sufficient. Goel and Singh [7] postulated that the “adequacy of transesophageal imaging possibly depends on the extent and the density of calcification,” and that, “there may have been some less dense areas through which a satisfactory window could probably be obtained.” It is also important to note that fluoroscopic evaluation of the chest is more sensitive to the presence of calcium than plain films [8], however, CT is superior to both fluoroscopy and plain films. 

Calcification of the left atrial wall or appendage or both constitutes a major complication and risk to mitral valve surgery due to difficulty in entering the left atrium, potential embolization, and impaired hemostasis [5]. The accepted surgical treatment for left atrial calcification is a total endoatrioectomy with mitral valve replacement [2]. This is preferred because in most cases the calcification does not extend beyond the endocardium. The procedure requires access to the left atrium through the interatrial groove. Possible complications during cardiac surgery include dislodgement of thrombi, which results in cerebral embolism and uncontrollable hemorrhage if the left atrium is entered through the calcified region because of LA wall rigidity [3]. The interatrial septum then serves as the surgical cleavage plane, since this area is noncalcified and is therefore exploited in order to prevent embolization and hemorrhage when a thrombus is present [2]. One problem encountered includes closure of the atriotomy, as it is difficult to suture calcified structures [4]. 

Two specific imaging findings need to be taken into account prior to any surgery, as they may be contraindicatory to proceed with surgery. First, the presence of a coconut atrium (calcified interatrial wall) will significantly complicate surgery and increase mortality [2]. Second, mitral annular calcification may progress to liquefaction necrosis, referred to as “caseous necrosis” which can make mitral valve repair very difficult and will also increase mortality [9, 10].

## 4. Conclusion

Porcelain atrium is a rare entity. Familiarity with the radiographic features of this unique distribution of calcium, in the correct clinical setting, will allow prompt recognition when interpreting chest X-rays and CT images. Mitral valvular calcification is commonly associated, as well as a history of rheumatic heart disease. Calcification of the interatrial septum and/or caseous necrosis of the mitral annulus could be potential contraindications for mitral valve surgery. Neither was present in our patient.
